# Biogenic trypanocidal sesquiterpenes: lead compounds to design future trypanocidal drugs - a mini review

**DOI:** 10.1186/2008-2231-21-35

**Published:** 2013-05-15

**Authors:** Soodabeh Saeidnia, Ahmad Reza Gohari, Azita Haddadi

**Affiliations:** 1 Medicinal Plants Research Center, Faculty of Pharmacy, Tehran University of Medical Sciences, PO Box 14155–6451, Tehran, Iran; 2 College of Pharmacy and Nutrition, University of Saskatchewan, Saskatoon, Canada

**Keywords:** Sesquiterpene, Trypanosomiasis, Treatment, Elatol, Lactones

## Abstract

Human trypanosomiasis is a parasitic disease among poor people in Africa and Latin America. Therapy against African and American trypanosomiasis is based on a few drugs that often cause severe side-effects. Therefore, it is essential to develop drug discovery especially from natural origins. Sesquiterpenes, a diverse group of natural terpenoids, are found in essential oils of many plants and show a broad range of bioactivities. They act through multiple mechanisms in the chemotherapy of trypanosomiasis. Some of these active compounds contain hydroperoxides, aldehydes, alcohols, α,β-unsaturated γ-lactone and even halogenated moieties. Among the compounds reported, sesquiterpene lactones showed a potent anti-trypanosoma effect comparable with commercial trypanocidal drugs. Trypanocidal activity of sesquiterpene lactones mostly depends on the reaction between γ-lactone moieties and nucleophile groups of trypanithione, which is essential for *Trypanosoma* defense against the oxidative stresses. Elatol is a sesquiterpenoid from marine algae, with a different structure and considerable trypanocidal activity which could be an interesting candidate for further antiprotozoal investigations. To develop novel drugs with higher efficacy and lower toxicity from natural products, this review summarizes the more recent information on trypanocidal activities of various sesquiterpenes.

## Introduction

### Trypanosomiasis and treatments

Trypanosomiasis is a widespread protozoan disease that mainly affects poor and marginal population in Latin America and Africa, and has been disregarded by the pharmaceutical industries and governments [1]. American Trypanosomiasis, well known as Chagas disease, is caused by *Trypanosoma cruzi* and starts with an acute phase then a chronic latent stage resulting in heart or gastrointestinal diseases. When the disease remains for decades, the infected people will be capable of transmitting it to the others [2,3]. Anti-chagastic drugs, such as nifurtimox or benznidazole, are currently recommended to treat the disease. Unfortunately, these agents have shown significant side effects with no benefits in established cardiac or gastrointestinal complications of chronic disease. Also there are some problems associated with these drugs in terms of long period of treatment, toxicity and even cost in endemic regions [4,5]. Sleeping sickness is another kind of trypanosomiasis (Human African Trypanosomiasis) and transmitted by the bite of the ‘Glossina’ insect, commonly known as the tsetse fly. *T. brucei rhodesiense* and *T. brucei gambiense* are responsible for East and West African trypanosomiasis respectively. Unfortunately, the current useful drugs have been discovered some decades ago, and also they are found as ordinary toxic to human as well as limited effectiveness. Eflornithine is the only effective medication and one of the most recently developed drugs against African Trypanosomiasis caused by *T. b. Gambiense*. But this medication is expensive for the patients residing in African developing countries [6,7]. However, monotherapy of patients with eflornithine exhibited adverse effects. On the other hand, the nifurtimox-eflornithine combination is much better therapy for late-stage of *T. b. Gambiense* trypanosomiasis. The mechanism of action for suramin (polysulfonated naphthylamine derivative of urea) is through inhibition of trypanosoma enzymes and growth factors. Melarsoprol is another choice employed in the late stage especially for CNS disorders of African trypanosomiasis. However, melarsoprol showed toxicity in 4-6% of patients. Eflornithine, which is available via World Health Organization, is less toxic and better tolerated compared to arsenic drugs. Pentamidine is usually used for early stages of African trypanosomiasis and strongly bounded to spleen, liver, and kidney. It does not pass through the blood–brain barrier sufficiently, so that is not able to treat CNS infection [8-11]. These reasons cause a priority for discovery of new trypanocidal compounds.

### Natural products as anti-trypanosoma agents

Literature review showed that there are many metabolites from plants or marine sources which have been observed highly active against *Trypanosoma* parasites. The active compounds mostly belong to phenolics and flavonoids (*e.g.* quercetin, catechin and apigenin), xanthones, dibenzofuranones, anthraquinones and alkaloids (*e.g.* actinodaphnine, cassythine and dicentrine). Interestingly, there have been many terpenic compounds reported as the potent trypanocidal agents. This group of natural products comprises triterpenes (*e.g.* ursolic acid and oleanolic acid), diterpenes (*e.g.* komaroviquinone and dracocephalone A), sesquiterpenes (*e.g.* zaluzanin D, dehydrocostus lactone and neurolenin B) and finally monoterpenes (*e.g.* monoterpene hydroperoxides and aldehydes) [12-16].

### Sesquiterpenes

Sesquiterpenoids are introduced as the section of compounds consisting of 15 carbon, which are derived from three isoprenoid units and distributed mainly in higher plants. Sesquiterpenes and monoterpenes are the important constituents of volatile oils in the essential oil bearing plants. Sesquiterpenes are the most diverse group of isoprenoids with approximately 5000 reported natural compounds, which can act as pheromones and juvenile hormones in plants. All sesquiterpenes are derived from FDP (farnesyl diphosphate), and the structural diversity of this class is greater than that of the monoterpenes. This could be attributed to the higher number of cyclizations possible from a precursor with five additional carbon atoms (over 7,000 for the sesquiterpenes compared to 1,000 for the monoterpenes) [17,18].

Besides a wide range of biological activities, sesquiterpenes have shown to be the potent trypanocidal compounds in some *in vitro* and *in vivo* systems. Therefore, in this review we focus on the trypanocidal activity of various types of recently isolated sesquiterpenes which might be a guide to find the lead compounds in treatment of both American and African trypanosomiasis. Although a few reviews have been published on natural trypanocidal compounds [19,20], there is no report on the classification of the active sesquiterpenes (in relation to their activity) against various *Trypanosoma* intermediates such as epimastigotes, trypomastigotes or amastigotes. We have previously published the first part of “Trypanocidal terpenes: Lead compounds to design future trypanocidal drugs” with the focus on trypanocidal monoterpenes [21].

### Trypanocidal sesquiterpenes

#### Sesquiterpene hydroperoxides

**Figure 1:**
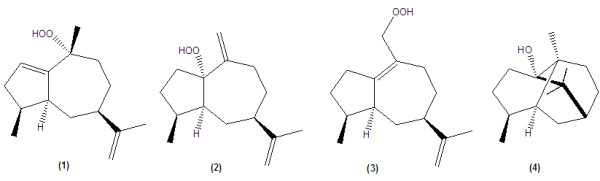
Chemical structures of trypanocidal sesquiterpene hydroperoxides.

In 2004, Kiuchi *et al*. reported the trypanocidal sesquiterpenes isolated from *Pogostemon cablin* (Lamiaceae) by the activity-guided fractionation of the acetone extract. Three new sesquiterpene hydroperoxides, 10α-hydroperoxyguaia-1,11-diene (1), 1α-hydroperoxy-guaia-10(15),11-diene (2) and 15α-hydroperoxy-guaia-1(10),11-diene (3), as well as a known terpenoid, patchouli alcohol (4) were reported. *In vitro* minimum lethal concentrations (MLC) of the hydroperoxides 1–3 against epimastigotes of *T. cruzi* were 0.84 μM (1), 1.7 μM (2) and 1.7 μM (3). The activity of the corresponding alcohols and patchouli alcohol was very weak (MLC > 200 μM) [18]. See the Figure 1.

### Sesquiterpene lactones

Sesquiterpene lactones are a group of bioactive substances consisting of different and a broad spectrum of sesquiterpenes which have been recognized in a number of plant families such as Acanthaceae, Apiaceae, Lauraceae, Magnoliaceae, Rutaceae, and the greatest numbers are found in the Asteraceae with over 3000 reported different structures [22].

Recently, Sulsen *et al*. has reported the trypanocidal activity of the sesquiterpene lactone psilostachyin C (5) which has been separated from a plant named *Ambrosia scabra* (Asteraceae) by bioassay-guided fractionation. There is evidence that *A. scabra* has been traditionally applied against intermittent fevers and worm infections. The isolated compound exhibited *in vitro* trypanocidal activity against *T. cruzi* epimastigotes, trypomastigotes and amastigotes and with a 50% inhibitory concentration (IC_50_) values of 0.6, 3.5 and 0.9 μM, respectively, with a 50% cytotoxic concentration (CC_50_) of 87.5 μM against mammalian cells. They mentioned that psilostachyin C showed a remarkable decrease in the number of circulating *T. cruzi* in mice after therapy with this substance during five days in comparison of control mice (received no treatment) (7.4 ± 1.2 × 10^5^ parasites/mL *vs.* 12.8 ± 2.0 × 10^5^ parasites/mL) [23]. Further studies on this compound (isolated from *A. tenuifolia*) showed that its anti-parasite activity is not reversible at concentrations higher than 1.0 μM. Additionally, its activity may inhibit at least in part by using glutathione. Ultrastructure study by transmission electron microscopy showed that psilostachyin was able to induce changes on the parasites (at level of 0.5 μM) like considerable mitochondrial swelling and the kinetoplast deformation [24].

In another study, Jimenez-Ortiz *et al.*, has recently reported the sesquiterpene lactones, mexicanin (6) and helenalin (7) as the active compounds against *T. cruzi*. Both compounds showed toxicity against parasite (IC_50_, 3.8 ± 0.19 and 1.9 ± 0.08 μM, respectively) compared to benznidazole (IC_50_, 8.6 ± 2.5 μM). The authors revealed that epimastigotes were less sensitive than trypomastigotes to the compounds whose activities were irreversible by using dithiotreitol as a reducing agent. The results obtained from that study showed that the mechanism of actions for these lactones seem different with that of the related lactone, dehydroleucodine (8) [25]. Dehydroleucodine was isolated from *Artemisia douglassiana* (Asteraceae) and reported to be active against the epimastigotes of *T. cruzi* at concentrations between 5 and 50 μM. Concentrations between 25 and 50 μM were found to be lethal for the parasites, whereas at lesser concentrations (between 5 and 10 μM) most of the cells remained alive for at least 96 h. This effect was also observed to be irreversible [26]. It has been reported that the dichloromethane extract of the aerial parts of *Eupatorium perfoliatum* (Asteraceae) exhibited anti *T. brucei* and *T. cruzi* activities under *in vitro* conditions (IC_50_, 14.0 and 53.6 μM, respectively) [27]. Biologically guided fractionation resulted in isolation of a dimeric guaianolide, *5S,6R,7R,8R,11R,14S-14-hydroxy-2-oxo-14(4S,5S,6R,7R,8R,11R-8-tigloyloxyguaia-1(10),2-diene-6,12,2,14-diolid-4-yl)-8-tigloyloxyguaia-1(10),3-diene-6,12-olide* (9) which was potent against *T. brucei* (IC_50_, 4.5 μM) and *T. cruzi* (IC_50_, 21.7 μM) but also exhibited a higher cytotoxicity (Selectivity Index (SI) = IC_50_(cytotoxic)/IC_50_(antiprotozoal), 2.6 and < 1, respectively). Another effective sesquiterpene was reported as: *5S,6R,7R,8R,11R-(−)-2-oxo-8-tigloyloxyguaia-1(10),3-diene-6,12-olide-14-carboxylic acid* (10) which showed activity against *T. brucei* (IC_50_, 7.7 μM) and *T. cruzi* (IC_50_ > 90.0 μM) but their selectivity index were not considerable. Another sesquiterpene was identified as: *3α,14-dihydroxy-8β-tigloyloxy-6βH,7αH,11αH-germacra-1(10)Z,4Z-dien-6,12-olide* (11). The latter compound showed moderate activity against *T. brucei* (IC_50_, 11.3 μM) [27].

*Neurolaena lobata* (Asteraceae) has been employed in traditional medicine of Guatemala, and it is reported that this plant contains three sesquiterpene lactones effective against trypomastigotes of *T. cruzi.* Literature revealed the isolation of a pure germacranolides, neurolenin B (12) and a mixture of the isomeric derivatives neurolenin C (13) and D (14) [28]. The sesquiterpenes exhibited high activity against both life stages of the parasite. Compounds 12–14 could inhibit the growth and development of trypanosome in the same range as nifurtimox and benznidazole (positive controls) would do. The activity of compound 12 was observed to be 2-fold more against epimastigotes (IC_90_, 6.3 ± 0.25 μM) and trypomastigotes (IC_90_, 4.9 ± 0.42 μM), compared to the mixture of compounds 13 and 14 (IC_90_, 11.7 ± 0.99 and 6.1 ± 0.60 μM, for epimastigotes and trypomastigotes, respectively).

**Figure 2:**
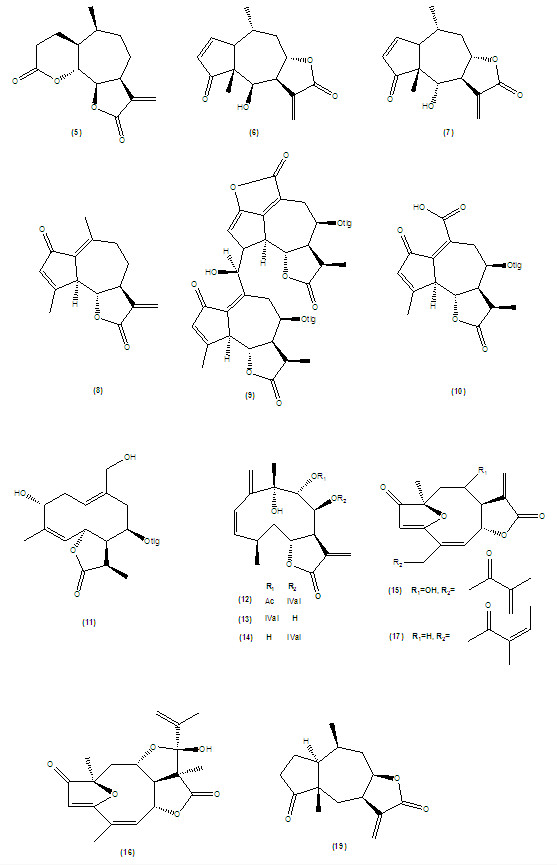
Chemical structures of trypanocidal sesquiterpene lactones.

Oliveira *et al*. (1996) reported the trypanocidal activity of the sesquiterpenes isolated from *Lychnophora passerine, L. pinaster* and *L. hichocarpha* against the trypomastigote forms of *T. cruzi*[19]. The sesquiterpene lactones goyazensolide (15), eremantholide C (16), and lychnopholide (17) have caused the complete lyses of the Y strain of *T. cruzi* (MIC, 240, 150 and 3600 μM, respectively). They reported that lychnopholide (17) had less activity on both Y and CL strains of *T. cruzi*[29]. Please see Figure 2 for chemical structures of sesquiterpene lactones.

### Secoaromadendrane type sesquiterpene

**Figure 3:**
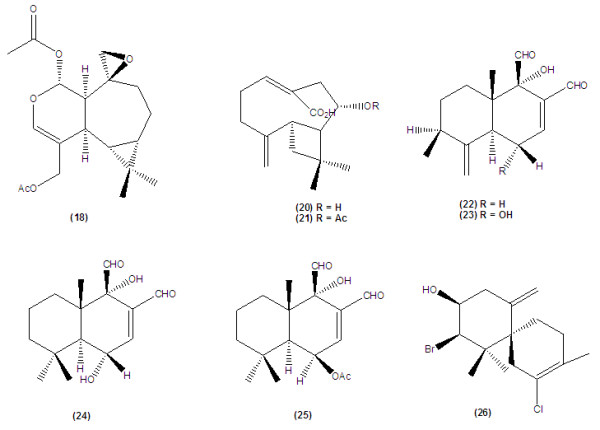
Structures of various trypanocidal secoaromadendrane, caryophyllene, drimane and coloratane aldehyde types together with elatol.

More recently, one *ent*-2,3-secoaromadendrane-type sesquiterpenoid, plagiochiline A (18), as well as a pseudoguaianolid, confertin (19), isolated from *Plagiochila disticha* and *Ambrosia peruviana,* have been evaluated against *T. cruzi* trypomastigotes. The authors revealed that although the isolated compounds showed considerable trypanocidal activities (IC_50_, 14.8 and 13.2 μM, respectively), they were highly cytotoxic against both human tumour cell lines and normal African green monkey kidney epithelial cells (VERO). GI_50_ (the concentration of a tested compound that causes 50% growth inhibition) on VERO cell line have been reported as 4.2 and 6.0 μM, respectively [30]. See Figure 3.

### Caryophyllene type sesquiterpenes

Jordao *et al*. (2003) reported that the ethyl acetate extract of the above ground parts of *Lychnophora salicifolia* (Asteraceae) indicated a significant trypanocidal activity against trypomastigote of *T. cruzi*, which was due to the presence of sesquiterpenoids lychnopholic acid (20) and acetyl lychnopholic acid (21), at least in part. The activities of these two sesquiterpenes were not noteworthy (IC_50_, 449.6 and 1029.0 μM, respectively) against *T. cruzi* trypomastigote compared to the positive control gentian violet (IC_50_, 76.0 μM). The authors pointed that the mixture of isolated compounds showed better activity owing to their synergic effects [31]. See Figure 3.

### Drimane and coloratane aldehyde sesquiterpenes

Recently, Wube and co-authors (2010) reported trypanocidal activity of the sesquiterpenes separated from *Warburgia ugandensis* Sprague (Canellaceae) against *T. brucei rhodesiense*[32]. *W. ugandensis* is growing in the East African and applyed as a trypanocidal medicinal plant. They mentioned that among the isolated substances examined for anti-trypomastigote activity, both drimane and coloratane sesquiterpenes (22–25) were identified as the highest anti-trypanosomal active compounds (IC_50_, 0.56-6.4 μM). The characteristic point of view for these substances is the presence of aldehyde functional groups at positions 8 and 9. See Figure 3.

### Sesquiterpene from marine source

The *Laurencia* complex *Lamouroux* (Rhodophyceae) comprises the most secondary metabolites in the marine environment which are predominantly sesquiterpenes like elatol (26) and are generally remarked by identification of halogen atoms in their molecular formula [33]. See Figure 3.

The *in vitro* trypanocidal activity of the compound elatol isolated from the Brazilian red seaweed *Laurencia dendroidea* has been recently reported using electron microscopy [34]. Elatol exhibited anti-parasite activity on the epimastigote, trypomastigote, and amastigote forms (IC_50_, 45.4, 1.38, and 1.01 μM, respectively) in a dose dependent manner. Furthermore, this compound showed no activity on the red blood cells with CC_50_ value of 27.0 μM for LLCMK2 cells. Aberrant-shaped cells, breaks in the plasma membrane, prominent swollen mitochondria, and extensive formation of cytoplasmic vacuoles have been reported in all the forms [32].

### Structure – anti- typanosoma activity relationships

Trypanocidal activity of the first group (sesquiterpene hydroperoxides) described in this review seems to be ascribable to the hydroperoxy functional group. This is attributed to the inactivity of the related alcohols similar to that of monoterpene hydroperoxide [35]. It is found that hydroperoxy group oxidates the glutathione, pyruvic and alpha-ketoglutaric acids, so that the oxidative decarboxylation of pyruvic acid makes the compounds toxic [36]. On the other hand, the compounds (both mono- and sesquiterpenes) containing hydroperoxide functional groups are easily destroyed to alcohol for example during hydro-distillation of the essential oils [35].

Many sesquiterpene lactones, the second group in this review, containing an α,β-unsaturated γ-lactone moiety have exhibited trypanocidal activity. Recently, Schmidt and co-authors (2009), have reported a structure - antiprotozoal activity study on a group of 40 sesquiterpene lactones against some protozoa such as *T. brucei rhodesiense* and *T. cruzi* alongside L6 rat skeletal myoblast cells [37]. All the experiments were carried out *in vitro* and some compounds showed high activity, particularly against *T. brucei* (such as helenalin and its esters, IC_50_, 0.05-0.1 μM). The main and distinct factors to create a trypanocidal activity were reported as: α,β-unsaturated functional groups which seem necessary for other biological activities of sesquiterpene lactones as well. Uchiyama (2009) suggested that trypanocidal activity of these compounds are mostly dependent on the covalent bond formation between γ-lactone moiety and nucleophiles (reaction with –SH group of trypanithione), which is essential for trypanosome living against the oxidative stresses [19]. It has been suggested that the irreversible trypanocidal activity of some sesquiterpenes such as dehydrocouledine (8) might be due to the above-mentioned mechanism since it is found that the reducing agents such as thiols can block the activity [26].

The characteristic α, β-unsaturated carbonyl group together with a trans-decalin ring system are the main structural features of the drimanes and coloratanes, except a methyl group at position 4 (in drimanes) which is shifted to position 3 in coloratane while leaving an exocyclic methylene group. Literature shows that drimane and coloratane sesquiterpenes, having two aldehydes (compounds 22–25), indicated higher activity against *T. brucei* than the corresponding sesquiterpene lactones. It is suggested that the dialdehyde functional groups would be hallmarked for trypanocidal activity [32]. It was also observed in monoterpene aldehydes that the compounds possessing aldehyde group were the strong trypanocidal active metabolites against both *T. cruzi* and *T. brucei*. The mentioned monoterpenes have a C-C double bond conjugated with the carbonyl group, which is well known as α, β–unsaturated aldehydes [21,38-40]. But as far as we could ascertain, there is a correlation between trypanocidal or cytotoxic activities of aldehyde-possessing compounds and their molecular structures [21]. Sesquiterpenes containing aldehydes are able to form covalent connection to amino moiety of proteins and inactivate them. In *Trypanosoma* parasite, they can form aldehyde-thiol adducts with sulphur containing components. Through the same mechanism, decrease in buffering agents can result in raising oxidative stress in both trypanosome and human cells [41].

**Figure 4:**
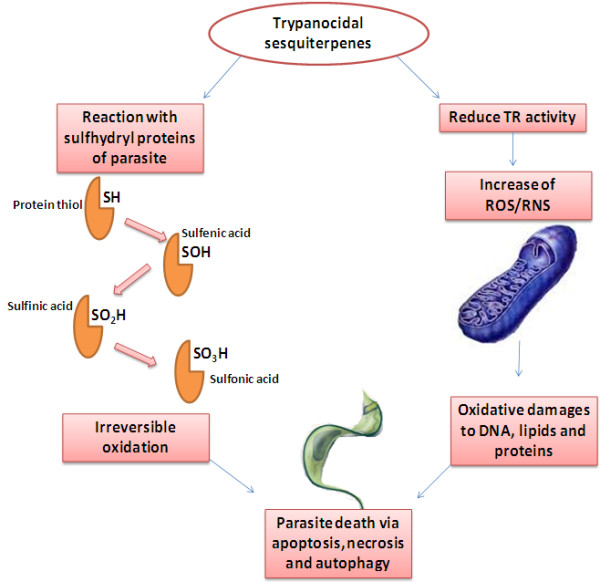
A schematic mechanism of action for trypanocidal sesquiterpenes.

On the basis of the literature, elatol (26), the major constituent of a red seaweed *L. dendroidea* showed significant activities against epimastigote, trypomastigote and amastigote forms of *T. cruzi*. Elatol has indicated a dose-dependent activity against the epimastigotes after 96 h of treatment. Moreover, elatol could destroy the trypomastigotes (IC_50_, 1.38 ±0.15 μM), and intracellular amastigotes (IC_50_, 1.01 ± 0.65 μM). The results were considerable in comparison of the reference drug, benznidazole (IC_50_, 24.3 μM) [33]. Literature revealed that this compound could not influence on the red blood cells (CC_50,_ 27.0 μM for LLCMK2 cells) and probably affected on particular metabolic pathway of trypanosoma. Alongside this effect, elatol is reported to be a potent antiproliferative agent against *Leishmania amazonensis*[42]. In fact, therapeutic activity of elatol involves mitochondria as the primary target leading to increase of reactive oxygen species (ROS) generation via the electron transport chain, which influence on cell membrane and DNA integrity and finally death of parasite [43,44]. For more details on mechanism of action for most of sesquiterpenes, please see Figure 4.

Additionally, the IC_50_ for the sesquiterpenes mentioned in this review are summarized in Table 1.

**Table 1:** **The related MIC and/or IC**_**50**_**of the active sesquiterpene compounds against*T. cruzi*and*T. brucei***

**Compounds**	**MLC ( μM)**	**IC**_**50**_**( μM)**	**IC**_**50**_**( μM)**	**References**
	***T. cruzi***	***T. cruzi***	***T. brucei***	
1	0.84			[18]
2	1.7			[18]
3	1.7			[18]
4	>200			[18]
5		0.6(E); 3.5(T); 0.9(A)		[23]
6		3.8 ± 0.19 parasite		[25]
7		1.9 ± 0.08 parasite		[25]
9		21.7(T)	4.5(T)	[27]
10		>90.0(T)	7.7(T)	[27]
11			11.3(T)	[27]
12		6.3 ± 0.25(E)*		[19]
		4.9 ± 0.42(T)*		
13,14		11.7 ± 0.99(E)*		[19]
		6.1 ± 0.60(T)*		
15	240**			[29]
16	150**			[29]
17	3600**			[29]
18		14.8(T)		[30]
19		13.2(T)		[30]
20		449.6(T)		[31]
21		1029.0(T)		[31]
22			0.56(T)	[32]
23			1.25(T)	[32]
24			0.64(T)	[32]
25			6.4(T)	[32]
26			45.4(E);1.38(T);1.01(A)	[32]

*IC_90_; ** MIC. A: amastigote; E: epimastigote; T: trypomastigote.

## Conclusion

Sesquiterpenes are known to be the aromatic compounds in the essential oils isolated from higher plants or marines. The medicinal plants containing these compounds have been consumed in traditional medicine for therapy of inflammation and known to possess a broad spectrum of biological activities such as antimicrobial, cytotoxic and antiviral agents. Literature review showed some reports on the anti-trypanosoma activity of sesquiterpenes. Some of these active compounds contain hydroperoxides, alcohols, α,β-unsaturated γ-lactone and even halogenated moieties. Among the compounds reported, sesquiterpene lactones demonstrated a potent anti-trypanosoma effect with an adequate selectivity index comparable with trypanocidal drugs. Trypanocidal activity of these compounds is mainly depended on the covalent bond formation between γ-lactone moieties and –SH group of trypanithione, which is essential for trypanosome in the oxidative stress conditions. Elatol is a sesquiterpenoid from marine algae with different structure which showed a relatively high trypanocidal activity and could be an interesting candidate for further antiprotozoal investigations.

## Competing interests

The authors declare that they have no competing interests.

## Authors’ contributions

SS: conception and design; ARG: drafting and revising the article; AH: preparing all data and articles used in the review. All authors read and approved the final manuscript.
